# Cancellation of outpatient appointments in patients with attention-deficit/hyperactivity disorder

**DOI:** 10.1371/journal.pone.0260431

**Published:** 2021-11-19

**Authors:** Kensuke Nomura, Ryosuke Tarumi, Kazunari Yoshida, Mitsuhiro Sado, Takefumi Suzuki, Masaru Mimura, Hiroyuki Uchida

**Affiliations:** 1 Department of Child Psychiatry, Shimada Ryoiku Medical Center for Challenged Children, Tokyo, Japan; 2 Department of Neuropsychiatry, Keio University School of Medicine, Tokyo, Japan; 3 Pharmacogenetics Research Clinic, Centre for Addiction and Mental Health, Toronto, Ontario, Canada; 4 Azrieli Adult Neurodevelopmental Centre, Centre for Addiction and Mental Health, Toronto, Ontario, Canada; 5 Department of Psychiatry, University of Toronto, Toronto, Ontario, Canada; 6 Department of Neuropsychiatry, University of Yamanashi Faculty of Medicine, Yamanashi, Japan; Tokyo Metropolitan Institute of Medical Science, JAPAN

## Abstract

**Background:**

Regular visit to psychiatric clinic is essential for successful treatment of any psychiatric condition including attention-deficit/hyperactivity disorder (AD/HD). However, cancellation of outpatient appointments in patients with AD/HD, which represents a significant medical loss, has not been systematically investigated to our knowledge.

**Methods:**

A systematic chart review was conducted for patients visiting the Shimada Ryoiku medical Center for Challenged Children in Japan at the age of ≤15 years from January to December 2013. The primary outcome measure was the cancellation rate, defined as the number of missed visits divided by the number of scheduled visits. The cancellation rates during 24 months after the first visit were compared between outpatients with AD/HD and other psychiatric disorders, including pervasive developmental disorders (PDD), and developmental coordination disorders and/or communication disorders (DCD-CD). A generalized linear model with binomial distribution was used to examine factors associated with cancellation rates exclusively in the AD/HD group.

**Results:**

We included 589 patients (mean ± SD age, 5.6 ± 3.4 years; 432 males) in the analysis. The cancellation rate in patients with AD/HD was 12.3% (95% confidence interval [CI]: 10.0–15.1), which was significantly higher than in those with PDD (5.6%, 95% CI: 3.8–8.3) and DCD-CD (5.3%, 95% CI: 3.6–7.8). Prescriptions of osmotic-release oral system-methylphenidate (OROS-MPH) and antipsychotics were associated with fewer cancellations in AD/HD patients (odds ratios: 0.61, 95% CI: 0.39–0.95 and 0.49, 95% CI: 0.25–0.95, respectively), although these significances did not find in the subgroup analysis including only patients with ≥ 6 years old.

**Conclusions:**

Patients with AD/HD were more likely to miss appointments compared to those with other psychiatric disorders. The impact of AD/HD medications as well as potential psychiatric symptoms of their parents or caregivers on appointment cancellations needs to be evaluated in more detail in future investigations.

## Introduction

Attention-deficit/hyperactivity disorder (AD/HD) is the most common childhood-onset neurodevelopmental disorder and is characterized by persistent inattention, hyperactivity, and impulsivity, resulting in various degrees of functional impairment [1–3]. The prevalence of this disorder among school-aged children is estimated to range from 3–12% [4, 5]. AD/HD seriously compromises the quality of life of patients and their caregivers. AD/HD has a comparable negative impact on the patients’ quality of life like other severe physical disorders, such as pediatric cancer and cerebral palsy [6]. The economic burden of AD/HD is also substantial. A systematic review showed that the average total costs of AD/HD ranged from €9,860 to €14,483 per patient and annual national costs were reported to be between €1,041 and €1,529 million in Europe [7]. In the United States, the overall national annual incremental costs of AD/HD were estimated to range from $143 to $266 billion (Doshi et al., 2012). While the majority of these costs was attributed to adults ($105–194 billion) compared with children/adolescents ($38–72 billion) [8] (Doshi et al., 2012), a student with AD/HD incurs an average annual incremental cost to the society of $5,007 as compared to $318 for a student in the comparison group [9].

A systematic review that included studies until 2011 reported untreated AD/HD was associated with poorer long-term self-esteem and social function outcomes compared with non-AD/HD controls [1]. More specifically, untreated AD/HD showed a higher percentage of poorer self-esteem and social function compared to non-AD/HD controls (57% vs 43% for self-esteem and 73% vs 27% for social function, respectively). Notably, pharmacological, non-pharmacological, and multimodal treatments contributed to favorable treatment outcomes (i.e., improvement of self-esteem and social function) [1]. With regard to pharmacological treatment, greater treatment adherence is positively correlated with better academic performance of patients with AD/HD. A study using academic records from Philadelphia public schools showed that better adherence to stimulants was associated with a significant improvement in academic grade point average (GPA) [10]. The mean GPA was significantly higher during stimulant-adherent than during stimulant-non-adherent marking periods (2.18 vs. 1.99, p < 0.0001). In addition, a regression coefficient representing the within-student association between stimulant adherence and GPA over time was 0.108 [10]. As there are several effective pharmacological and non-pharmacological treatment options for AD/HD [11], these findings emphasize the need for greater adherence to treatment to achieve better outcomes for patients with AD/HD. However, patients with AD/HD do not regularly visit their clinic in part because of their intrinsic symptoms such as inattention. However, the issue of missed or canceled appointments has not been systematically investigated to the best of our knowledge.

To fill this gap in the literature, we conducted a systematic chart review of the cancellation rates among patients with AD/HD at a specialized children’s hospital in Tokyo, Japan, in comparison to patients with other pediatric illnesses. We also examined factors associated with better attendance at the clinic in patients with AD/HD.

## Methods

### Patient population

A retrospective medical chart review was conducted at the Shimada Ryoiku Medical Center for Challenged Children, a facility mostly dedicated to mental health issues among youths in Tokyo, Japan. Patients who visited this center for the first time between January 1, 2013 and December 31, 2013 were screened. We selected the subject’s data in 2013 as the most recent data that could be considered as “stable” in terms of diagnostic criteria and pharmacotherapy, taking into account that the diagnostic criteria started the transition from Diagnostic and Statistical Manual of Mental Disorders 4^th^ edition text revision (DSM-IV-TR) to DSM-5 in Japan in 2014 and that osmotic-release oral system-methylphenidate (OROS-MPH) and atomoxetine (ATX) were approved for the treatment of AD/HD in Japan in 2007 and 2009, respectively. Among them, outpatients were included if they were 15 years or younger at the time of the first visit and visited the center at least twice. The study was carried out in accordance with the latest version of the Declaration of Helsinki. It was approved by the Ethics Committee of Shimada Ryoiku Medical Center for Challenged Children and exempted from the requirement for informed consent because the study exclusively dealt with de-identified data acquired during routine care.

### Study design

The collected information included diagnoses according to the DSM-IV-TR for psychiatric disorders [12] and the International Statistical Classification of Diseases and Related Health Problems 10^th^ revision (ICD-10) [13] for physical illnesses, the dates of scheduled visits for 24 months after their first visit, the dates of actual visits during the period, age, sex, height, weight, marital status of their parents, family structure, family history of psychiatric conditions, and psychotropic medications prescribed for 24 months after their first visit. We confirmed the main diagnosis of each patient included in this study based on DSM-IV-TR and ICD-10 through the following process: 1) an initial diagnostic confirmation with multiple physicians in a case conference at the study site after the first visit, and 2) reconfirmation of the main diagnosis at discharge or after 24 months of the first visit through the discussion with attending physicians. The patients who met the following criteria were defined as having intellectual disability (ID; referred to as “mental retardation” in the DSM-IV-TR, which is not currently used as a diagnostic term) in this study: 1) those who were not diagnosed with Down syndrome (DS) or cerebral palsy (CP) and 2) those who had intelligence quotient or developmental quotient level were less than 70.

### Data analysis

**Fig 1:**
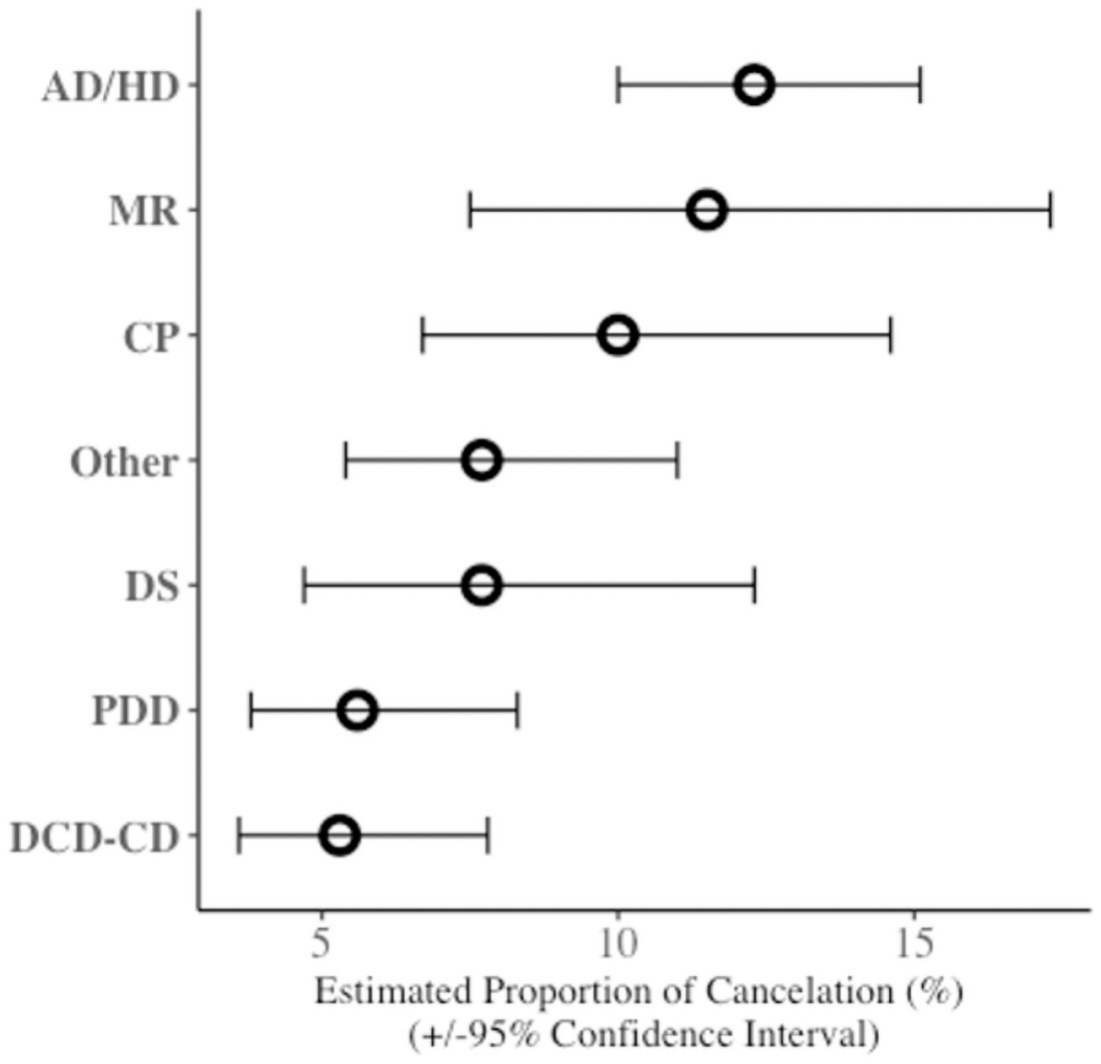
Cancellation rate in each diagnostic group. The cancellation rate in the AD/HD group was significantly higher than that in the PDD or DCD-CD groups. Open circles and error bars represent means and 95% confidence intervals of cancellation rate, respectively. The values were adjusted by age and sex. AD/HD attention-deficit/hyperactivity disorder; CP cerebral palsy; DCD-CD developmental coordination disorders and/or communication disorders; DS Down syndrome; ID intellectual disability; PDD pervasive developmental disorders.

Statistical analyses were performed using SPSS version 24.0 (IBM, Chicago). Fig 1 was created using the ggplot2 package in R (version 4.0.2). The primary outcome measure was the cancellation rate, defined as the number of missed visits divided by the number of scheduled visits. In the present study, cancellation was defined as no show-up to the outpatient clinic without prior notice or with notice on the appointed day or the day before.

A generalized linear model with binomial distribution adjusted for age and sex was used to compare cancellation rates among the following seven groups classified by their main diagnoses: AD/HD, pervasive developmental disorders (PDD), ID, developmental coordination disorders and/or communication disorders (DCD-CD), CP (ICD-10, G80.0–9), and DS (ICD-10, Q90.0–9). To simplify the analysis, patients meeting DSM-IV-TR criteria for developmental coordination disorder and communication disorders were combined in the DCD-CD group because they often co-exist. Post-hoc analyses were conducted to compare cancellation rates in the AD/HD group and those in each of the other groups. Another generalized linear model with binomial distribution was used to examine factors associated with cancellation rates exclusively in the AD/HD group with the following independent variables: age, sex, the number of family members living with the patient, and use of medications OROS-MPH, ATX, or antipsychotics. Subgroup analyses were performed in only patients who were ≥ 4 or 6 years old at the first visit. Only OROS-MPH and ATX were approved in Japan for treatment of AD/HD at the time this study was conducted. Antipsychotics were not approved for the treating irritability of patients with ASD in Japan when this chart-review was conducted. A p-value < 0.05 was considered statistically significant (two-tailed) and Bonferroni adjustments were applied where necessary.

## Results

### Characteristics of subjects

There were 624 subjects who visited the center for the first time between January 1, 2013 and December 31, 2013. Thirty-five of these patients did not appear on the second visit, leaving 589 patients to be included in the analysis. The average (± standard deviation) age was 5.6 ± 3.4 years and there were 432 males (73.2%). The mean number of reservations was 8.3 ± 5.4, while the mean number of cancellations was 0.7 ± 1.3 over the 24 months. The demographic and clinical characteristics of the patients are summarized in Table 1.

**Table 1:** Demographic and clinical characteristics of the patients included in the study.

Groups	All (N = 589)	AD/HD group^a^	PDD group^b^	CP group^c^	DCD-CD group^d^	ID group^e^	DS group^f^	Other group^g^
Characteristics								
Age, years	5.6 ± 3.4	7.6 ± 2.6	6.4 ± 3.3	3.4 ± 3.7	3.7 ± 1.3	5.6 ± 3.9	1.9 ± 2.1	5.6 ± 3.8
	(0–14)	(2–13)	(2–14)	(0–14)	(1–9)	(1–14)	(0–7)	(0–13)
Number of reservations^h^	4902	1766	705	431	787	271	372	570
	(8.32 ± 5.43)	(9.81 ± 6.13)	(6.47 ± 3.84)	(12.3 ± 8.03)	(6.50 ± 2.92)	(7.13 ± 3.36)	(10.9 ± 4.70)	(7.92 ± 6.06)
Number of cancellations^i^	419	207	38	38	38	30	26	42
	(0.71 ± 1.27)	(1.15 ± 1.59)	(0.35 ± 0.77)	(1.09 ± 1.54)	(0.31 ± 0.58)	(0.79 ± 1.32)	(0.76 ± 1.13)	(0.58 ± 1.31)
AD/HD score at school	N.A.	21.1 ± 11.7	N.A.	N.A.	N.A.	N.A.	N.A.	N.A.
AD/HD score at home	N.A.	24.2 ± 11.1	N.A.	N.A.	N.A.	N.A.	N.A.	N.A.
Number of family members living with the patient	3.0 ± 1.0	3.1 ± 1.0	2.9 ± 0.9	2.8 ± 0.8	2.9 ± 0.9	2.9 ± 0.9	2.9 ± 1.1	3.1 ± 1.1
Number of siblings	1.0 ± 0.8	1.1 ± 0.8	0.8 ± 0.8	0.8 ± 0.8	0.9 ± 0.8	0.9 ± 0.8	1.1 ± 0.9	1.1 ± 0.9
Sex								
Male	432 (73.3)	152 (84.4)	83 (76.1)	24 (68.6)	86 (71.1)	26 (68.4)	17 (50.0)	44 (61.1)
Family history of psychiatric conditions								
Yes	18 (3.1)	11 (6.1)	2 (1.8)	0 (0.0)	1 (0.8)	0 (0.0)	0 (0.0)	4 (5.6)
Parental divorce								
Yes	50 (8.5)	25 (13.9)	6 (5.5)	3 (8.6)	5 (4.1)	0 (0.0)	1 (2.9)	10 (13.9)
Bereavement of either parent								
Yes	1 (0.2)	0 (0.0)	0 (0.0)	1 (2.9)	0 (0.0)	0 (0.0)	0 (0.0)	0 (0.0)
Medication								
OROS-MPH	68 (11.5)	61 (33.9)	5 (4.6)	0 (0.0)	1 (0.8)	0 (0.0)	0 (0.0)	1 (1.4)
Atomoxetine	53 (9.0)	46 (25.6)	3 (2.8)	0 (0.0)	1 (0.8)	0 (0.0)	1 (2.9)	2 (2.8)
Antipsychotics	27 (4.6)	20 (11.1)	4 (3.7)	0 (0.0)	0 (0.0)	0 (0.0)	1 (2.9)	2 (2.8)
Antidepressants	5 (0.8)	0 (0.0)	0 (0.0)	1 (2.9)	0 (0.0)	1 (2.6)	0 (0.0)	3 (4.2)
Other psychotropics	11 (1.9)	6 (3.3)	1 (0.9)	1 (2.9)	0 (0.0)	0 (0.0)	0 (0.0)	3 (4.2)

Values are shown as mean ± S.D. (range) or n (%).

^a^ Patients with AD/HD were included in the AD/HD group (n = 180).

^b^ Patients with autism disorder, Asperger syndrome, and pervasive developmental disorder not otherwise specified patients were included in the PDD group (n = 109).

^c^ Patients with cerebral palsy and periventricular leukomalacia were included in the CP group (n = 35).

^d^ Patients with developmental coordination disorders and communication disorders were included in the DCD-CD group (n = 121).

^e^ Patients with intellectual disability were included in the ID group (n = 38).

^f^ Patients with DS were included in the DS group (n = 34).

^g^ The other patients were included in the other group (n = 72).

^h, i^ The values are shown as the sum (mean ± S.D.).

AD/HD, attention-deficit/hyperactivity disorder; CP, cerebral palsy; DCD-CD, developmental coordination disorders and/or communication disorders; DS, Down syndrome; ID, intellectual disability; N.A, not applicable; OROS-MPH, osmotic-release oral system-methylphenidate; PDD, pervasive developmental disorders; SD, standard deviation.

### Comparison of the cancellation rate between the AD/HD group and the other groups

The proportion of cancellations in each group, which was calculated as an estimated marginal proportion adjusted by sex and age, is shown in Table 2 and Fig 1. The cancellation rate in the AD/HD group was significantly higher than that in the PDD or DCD-CD groups (Table 3 and Fig 1). The cancellation rate in the AD/HD group was numerically higher than that in the other four groups, although the difference did not reach statistical significance (Table 3 and Fig 1).

**Table 2:** Cancellation rates in each diagnostic group.

Groups	AD/HD group^a^	PDD group^b^	CP group^c^	DCD-CD group^d^	MR group^e^	DS group^f^	Other group^g^
Cancellation rate (%)^h^	12.3 [10.0–15.1]	5.6 [3.8–8.3]	10.0 [6.7–14.6]	5.3 [3.6–7.8]	11.5 [7.5–17.3]	7.7 [4.7–12.3]	7.7 [5.4–11.0]

Values are shown as mean and 95% confidence intervals (%).

^a^ AD/HD patients were included in the AD/HD group (n = 180).

^b^ Patients with autism disorder, Asperger syndrome, and pervasive developmental disorder, and not otherwise specified patients were included in the PDD group (n = 109).

^c^ Patients with cerebral palsy and periventricular leukomalacia were included in the CP group (n = 35).

^d^ Patients with developmental coordination disorders and communication disorders were included in the DCD-CD group (n = 121).

^e^ Patients with intellectual disability were included in the ID group (n = 38).

^f^ Patients with DS were included in the DS group (n = 34).

^g^ The other patients were included in the other group (n = 72).

^h^ The values were adjusted for age and sex.

AD/HD, attention-deficit/hyperactivity disorder; CP, cerebral palsy; DCD-CD, developmental coordination disorders and/or communication disorders; DS, Down syndrome; ID, intellectual disability; PDD, pervasive developmental disorders.

**Table 3:** Comparison of cancellation rate between patients with AD/HD and the other groups.

	Difference in proportions	Standard error	Wald chi-square	Df	P-value
PDD group	-0.07	0.016	16.6	1	**< 0.001**
CP group	-0.02	0.024	0.09	1	1
DCD-CD group	-0.07	0.017	16.2	1	**< 0.001**
ID group	-0.01	0.028	0.07	1	1
DS group	-0.05	0.025	3.5	1	0.367
Other group	-0.05	0.02	5.46	1	0.117

Each group was compared with the AD/HD group.

Bonferroni adjusted p-values are shown.

P-values < 0.05 are shown in bold.

The values were adjusted for age and sex.

AD/HD. attention-deficit/hyperactivity disorder; CP, cerebral palsy; DCD-CD, developmental coordination disorders and/or communication disorders; DS, Down syndrome; ID, intellectual disability; PDD, pervasive developmental disorders.

### Factors associated with the cancellation rate in the AD/HD group

The use of OROS-MPH or antipsychotics was significantly associated with a lower cancellation rate in the AD/HD group. No other factors showed an association with the cancellation rate in this specific group (Table 4). However, only OROS-MPH was significantly associated with a lower cancellation rate in the AD/HD group in a subgroup analysis including only patients who were ≥ 4 years old at the first visit (S1 Table). None of the factors was significantly associated with a lower cancellation rate in another subgroup analysis including only patients who were ≥ 6 years old at the first visit (S2 Table).

**Table 4:** Association between characteristics and cancellation rate in the AD/HD patients included in this study.

Variables	Wald chi-square	Df	P-value	Exp (B)	95% Wald CI for Exp (B)	
					Lower	Upper
Age (years)	1.182	1	0.277	1.047	0.964	1.138
Sex						
Male	0.011	1	0.918	1.032	0.569	1.869
Female	1 (reference)					
Use of medications						
OROS-MPH Yes	4.734	1	**0.030**	0.608	0.389	0.952
OROS-MPH No	1 (reference)					
Atomoxetine Yes	0.698	1	0.403	0.818	0.510	1.311
Atomoxetine No	1 (reference)					
Antipsychotics Yes	4.435	1	**0.035**	0.489	0.251	0.952
Antipsychotics No	1 (reference)					
Family history of psychiatric conditions Yes	0.365	1	0.546	0.782	0.353	1.735
Family history of psychiatric conditions No	1 (reference)					
Number of family members	0.344	1	0.557	1.066	0.861	1.321

P-values <0.05 are shown in bold.

AD/HD, attention-deficit/hyperactivity disorder; CI, confidence interval; df, degree of freedom; Exp, exponential function; OROS-MPH, osmotic-release oral system-methylphenidate.

## Discussion

There are two main findings of the present study: (1) The cancellation of outpatient appointments without prior notice was approximately twice as frequent among patients with AD/HD as among those with PDD or DCD-CD, and (2) the prescription of OROS-MPH or antipsychotics was related to less frequent cancellations from patients with AD/HD. To our knowledge, this is the first study to show that patients with AD/HD cancel outpatient appointments more frequently than children with other psychiatric diseases and suggest that some psychotropic medications may protect against missed clinical appointments. The results are highly relevant for those involved in treatment as they need to be more vigilant with patients with AD/HD than other patients to keep timely appointments. Furthermore, potentially effective treatments could help facilitating regular follow-ups, which in turn could improve long-term outcomes for patients with AD/HD.

The fact that the cancellation rate was significantly higher in patients with AD/HD than in those with PDD or DCD-CD may be caused at least in part by inattention symptoms unique to AD/HD. However, given the mean age of the patients included was below six years old and they usually visited the hospital with their parents or caregivers, these patients should have been able to attend their appointments as scheduled if their parents or caregivers had remembered the appointments. This may imply that their parents or caregivers also suffered from some inattentive symptoms and therefore forgot their children’s appointments. This evidence highlights the importance of genetics in the pathogenesis of this condition. For example, a six-fold higher risk of AD/HD has been reported among children of mothers with a diagnosis of AD/HD [14]. The relatives of probands with AD/HD were identified to have at least a five times higher risk of having AD/HD. The risk in relatives with probands diagnosed with AD/HD according to the DSM-IV was 16%, while the risk in the relatives of healthy controls was 3% [15]. Likewise, the risk for attention deficit disorder according to the DSM-III has been reported to be higher among relatives than in healthy controls (25% vs. 4%) [15–18]. These findings collectively underscore the need for a treatment approach that targets not only children but also their parents to enhance treatment engagement. Although no significant effect of psychiatric family history on cancellation rates in the AD/HD group was found in this study, it should be noted that information on the presence/absence of the AD/HD diagnosis in parents or caregivers or the awareness of AD/HD symptoms in them, which could contribute to cancellation rates, was not collected in this study. Avoiding missed medical appointments is of high clinical relevance for a resource perspective. We may need to consider effective measures such as rigid reservation systems, reminder calls, or emails the day before or earlier, or setting cancellation fees.

The use of OROS-MPH and the prescription of antipsychotics were significantly associated with less frequent cancellation without notice among the children in the AD/HD group, while the use of ATX did not show any significant effect. This finding might reflect a quick mode of action for the medications. In other words, the earlier the drug is effective, the earlier the symptoms relapse upon discontinuation, and the more urgent the need to continue the drug would be felt. Such clinical scenarios may serve as a motivation of patients as well as their caregivers to go to the clinics as scheduled so as not to miss the medications. The onset of the effect of OROS-MPH is almost immediate. Armstrong et al. found that OROS-MPH was significantly more effective than placebo at 1, 2, 4, 10, 11, and 12.5 hours post-administration [19]. All antipsychotic medications for children administered during this observation period in Japan were drugs used off-label. In fact, the evidence for antipsychotics in the treatment of AD/HD is rather weak [11]. However, one double-blind randomized controlled study that used risperidone or MPH for preschool children with AD/HD found that both medications showed a significant improvement based on the parent AD/HD Rating Scale (p < 0.001) and Parent Conners Rating Scale (p < 0.001) over the six weeks of the treatment [20]. Another 6-week open-label study also reported that aripiprazole significantly improved AD/HD symptoms assessed using some measures including the AD/HD Rating Scale-IV in AD/HD patients aged 8–12 years old [21]. However, it should be noted that no significant effect of OROS-MPH and/or antipsychotics was found on cancellation rate in the AD/HD group in the subgroup analyses (S1 and S2 Tables), especially in the subgroup analysis including only patients who were ≥ 6 years old at the first visit. Compared to these drugs, the onset of the effect of ATX appears slower. The median time to improvement was 3.7 weeks, but remission of symptoms did not occur until a median of 14.3 weeks in an open-label ATX dosing trial in Canada [22]. Since it takes at least two weeks to reach an effective dose according to the official protocol of ATX administration in Japan, the results of this Canadian study appear cogent. In contrast, Buitelaar et al. showed that the risk of relapse after discontinuing stimulants, including OROS-MPH and Guanfacine extended-release, was substantially higher than that when ATX was discontinued [23] Additionally, Wernicke et al. reported that the incidence of discontinuation-emergent adverse events was low and there were no statistically significant differences between patients who abruptly discontinued ATX and those who continued placebo, indicating good acceptability [24]. Based on this reasoning, the use of ATX might not contribute to lower cancellation rates. However, the contribution of onset of action and withdrawal symptoms as well as the overall tolerability of the drug in keeping medical appointments would be a matter of further investigation.

There are several limitations to this study. First, the results of the present study should be interpreted in light of the relatively small sample size and the fact that all patients were Japanese and the site was a single hospital dedicated to mental health for the youth. Second, there was a lack of information on comorbidities, which might have an impact on the cancelation rate. This is because comorbidities were not discussed in detail in a case conference and sufficient information on comorbidities was not always available in the medical charts. Third, the subject’s data used in this study was relatively old (i.e., the data in 2013), although we selected the most recent data that could be considered stable in terms of diagnostic criteria and pharmacotherapy not to make the interpretation of study results complicated. Nevertheless, further investigation with newer data (e.g., data in the mid-2020s) will also be required to validate our findings. Fourth, there is lacking information on whether patients were offered specific programs for ADHD, including summer treatment programs, parent training, and social skills training. Fifth, information on some clinical and demographic characteristics, such as socioeconomic status and psychiatric diagnoses/psychiatric symptoms of their parents or caregivers was lacking. The lack of information on psychiatric diagnoses (e.g., ADHD) of their parents or caregivers may have had a significant impact on the findings in this study. For example, inattentive symptoms of the patients’ parents or caregivers could result in missing their children’s appointments. In addition, it was unclear why their parents or caregivers did not bring their children to the hospital as scheduled. Sixth, it should be noted that the main diagnosis was determined by a consensus of multiple physicians based on DSM-IV-TR and ICD-10; however, the diagnosis was determined by using the information collected thorough the assessment in clinical practice. In addition, any special diagnostic tools such as Conners 3rd edition [25] and Autism Diagnostic Interview-Revised [26] were not used. Seventh, there was no available data on the intelligence quotient for the patients except for those with ID, although the intelligence quotient level might affect the findings in this study. Finally, the retrospective nature of this survey should be acknowledged. The preliminary findings in this study need to be replicated in a larger number of representative samples, using a prospective study design.

## Conclusions

In summary, we found that children with AD/HD were more likely to cancel outpatient treatment appointments than children with other psychiatric conditions and that prescribing OROS-MPH and antipsychotics was associated with lower cancellation rates. Our results suggest that psychotropic medications such as OROS-MPH and antipsychotics may reduce appointment cancellations in patients with AD/HD who are more likely to miss appointments compared to other psychiatric disorders, although this result should be interpreted with caution given the negative findings in the subgroup analysis. These findings underscore the importance of family involvement and effective pharmacotherapy for the treatment of children with AD/HD.

## Supporting information

S1 Table Association between characteristics and cancellation rate in the AD/HD patients who were ≥ 4 years old at the first visit. (DOCX)

S2 Table Association between characteristics and cancellation rate in the AD/HD patients who were ≥ 6 years older at the first visit. (DOCX)

S1 Dataset Anonymized dataset of this study. (XLSX)
