# Supplementary material for: Cancellation of outpatient appointments in patients with attention-deficit/hyperactivity disorder
Source: PLoS One. 2021 Nov 19;16(11):e0260431. doi: 10.1371/journal.pone.0260431 (PMC8604341; doi:10.1371/journal.pone.0260431)
Supplement: S1 Table — (DOCX) [file pone.0260431.s001.docx]

**S1 Table. Association between characteristics and cancellation rate in the AD/HD patients** **who were** **≥ 4 years old at the first visit.**

| Variables | Wald chi-square | Df | P-value | Exp (B) | 95 % Wald CI for Exp (B) | |
| --- | --- | --- | --- | --- | --- | --- |
|  |  |  |  |  |  |  |
|  |  |  |  |  | Lower | Upper |
| Age (years) | 0.530 | 1 | 0.466 | 1.035 | 0.943 | 1.137 |
| Sex |  |  |  |  |  |  |
| Male | 0.000 | 1 | 0.998 | 0.999 | 0.523 | 1.908 |
| Female | 1 (reference) |  |  |  |  |  |
| Use of medications |  |  |  |  |  |  |
| OROS-MPH Yes | 4.363 | 1 | **0.037** | 0.615 | 0.390 | 0.971 |
| OROS-MPH No | 1 (reference) |  |  |  |  |  |
| Atomoxetine Yes | 0.774 | 1 | 0.379 | 0.807 | 0.500 | 1.302 |
| Atomoxetine No | 1 (reference) |  |  |  |  |  |
| Antipsychotics Yes | 4.435 | 1 | 0.065 | 0.525 | 0.295 | 1.042 |
| Antipsychotics No | 1 (reference) |  |  |  |  |  |
| Family history of psychiatric conditions Yes | 0.365 | 1 | 0.663 | 1.220 | 0.499 | 2.983 |
| Family history of psychiatric conditions No | 1 (reference) |  |  |  |  |  |
| Number of family members | 0.344 | 1 | 0.398 | 1.103 | 0.879 | 1.385 |

P-values <0.05 are shown in bold.

The numbers of the patients who received MPH and antipsychotics during the observation period were 60 and 19, respectively.

AD/HD, attention-deficit/hyperactivity disorder; CI, confidence interval; df, degree of freedom; Exp, exponential function; OROS-MPH, osmotic-release oral system-methylphenidate
