# Supplementary material for: Cancellation of outpatient appointments in patients with attention-deficit/hyperactivity disorder
Source: PLoS One. 2021 Nov 19;16(11):e0260431. doi: 10.1371/journal.pone.0260431 (PMC8604341; doi:10.1371/journal.pone.0260431)
Supplement: S2 Table — (DOCX) [file pone.0260431.s002.docx]

**S2 Table. Association between characteristics and cancellation rate in the AD/HD patients who were ≥ 6 years older at the first visit.**

| Variables | Wald chi-square | Df | P-value | Exp (B) | 95 % Wald CI for Exp (B) | |
| --- | --- | --- | --- | --- | --- | --- |
|  |  |  |  |  |  |  |
|  |  |  |  |  | Lower | Upper |
| Age (years) | 0.026 | 1 | 0.872 | 1.011 | 0.889 | 1.149 |
| Sex |  |  |  |  |  |  |
| Male | 0.021 | 1 | 0.884 | 1.052 | 0.529 | 2.094 |
| Female | 1 (reference) |  |  |  |  |  |
| Use of medications |  |  |  |  |  |  |
| OROS-MPH Yes | 1.881 | 1 | 0.170 | 0.708 | 0.432 | 1.160 |
| OROS-MPH No | 1 (reference) |  |  |  |  |  |
| Atomoxetine Yes | 1.797 | 1 | 0.180 | 0.707 | 0.426 | 1.174 |
| Atomoxetine No | 1 (reference) |  |  |  |  |  |
| Antipsychotics Yes | 3.826 | 1 | 0.050 | 0.484 | 0.234 | 1.001 |
| Antipsychotics No | 1 (reference) |  |  |  |  |  |
| Family history of psychiatric conditions Yes | 0.740 | 1 | 0.390 | 1.719 | 0.500 | 5.906 |
| Family history of psychiatric conditions No | 1 (reference) |  |  |  |  |  |
| Number of family members | 0.929 | 1 | 0.335 | 1.128 | 0.883 | 1.440 |

The numbers of the patients who received MPH and antipsychotics during the observation period were 54 and 18, respectively.

AD/HD, attention-deficit/hyperactivity disorder; CI, confidence interval; df, degree of freedom; Exp, exponential function; OROS-MPH, osmotic-release oral system-methylphenidate
